# Biosynthesis of Zinc Nanoparticles From Actinobacterium Streptomyces Species and Their Biological Potential

**DOI:** 10.7759/cureus.54124

**Published:** 2024-02-13

**Authors:** Aravind Sivakumar, Vasugi Suresh, Sathya Sethuraman, Pitchiah Sivaperumal

**Affiliations:** 1 Dentistry, Saveetha Dental College and Hospitals, Saveetha Institute of Medical and Technical Sciences, Saveetha University, Chennai, IND; 2 Physiology, Saveetha Dental College and Hospitals, Saveetha Institute of Medical and Technical Sciences, Saveetha University, Chennai, IND; 3 Prosthodontics, Saveetha Dental College and Hospitals, Saveetha Institute of Medical and Technical Sciences, Saveetha University, Chennai, IND

**Keywords:** gram-positive bacteria, gram-negative bacteria (gnb), nanoparticles, biosynthesis, antimicrobial, zinc nanoparticle, zone of inhibition, green synthesis, streptomyces, marine actinobacterium

## Abstract

Background: In today's world, antibiotic-resistant microorganisms are a major concern. There is solid evidence that metal nanoparticles (NPs) tend to have antimicrobial properties. The most effective substitute for antibiotic resistance is the incorporation of metal NPs. The antibacterial properties of NPs are currently being explored and shown to be successful. Zinc (Zn) NPs that are biosynthesized from marine Actinobacterium proved to be more biocompatible, bioactive, and affordable.

Aim: This study aims to investigate the synthesis of ZnNPs from Actinobacterium *Streptomyces *species and their antimicrobial effects against gram-positive and gram-negative bacteria.

Materials and methods: The current study uses natural, considerably safer processes to synthesize ZnNPs from marine Actinobacteria with little to no negative side effects. It involves sample collection, identification, and isolation of Actinobacterium *Streptomyces* species. The isolated sample was air-dried, and extracts of ZnNPs were taken. Among the isolates from marine sediment, two Actinobacteria that generate bioactive secondary metabolites-*Streptomyces* species (MOSEL-ME28) and *Rhodococcus rhodochrous* (MOSEL-ME29)-were selected for extracellular synthesis of ZnNPs. The antimicrobial activity of the biosynthesized ZnNPs from marine Actinobacteria was analyzed against *Staphylococcus *(MRSA), *Klebsiella pneumoniae*,* *and *Streptococcus mutans. *The results were statistically analyzed and graphs were created.

Results: ZnNPs obtained from Actinobacterium *Streptomyces* species exhibited antimicrobial effects against *Staphylococcus *(MRSA), *Klebsiella*,* and Streptococcus mutans*. At 280 nm wavelength, analysis of the UV spectrum showed a notable absorbance value of 1.8. The antibacterial efficacy against *Staphylococcus* MRSA, *Klebsiella* species, and *Streptococcus mutans* was assessed by measuring the zone of inhibition in diameter. The zones of inhibition were 8, 8, and 7 mm on the evaluation for* Streptococcus mutans*, *S. aureus*, and *Klebsiella* species, respectively, at a dose of 75 μg/mL. When the dosage was increased to 100 μg/mL, the inhibition zones were found to be 9.5, 9, and 7.5 mm for the respective bacterial strains.

Conclusion: ZnNPs are biosynthesized from marine Actinobacterium *Streptomyces *species in this research study. They have a significant antimicrobial activity against both gram-positive and negative bacteria. This indicates that ZnNPs have enormous antimicrobial potential and have an extensive spectrum of applications. However, clinical trials must be completed before it can be used safely on patients.

## Introduction

The synthesis of nanoparticles (NPs) has produced intriguing achievements at the newly developing interface of nanobiosciences [1]. Nanotechnology usually refers to the physical and scientific study of measurable particles between 1 and 100 nm in size. The reduced bulk has created more chances to use metallic materials and their mixtures in pharmaceuticals. NPs that are produced by conventional physical and chemical processes employ reducing and stabilizing chemicals which pose a fatal risk to both the earth and the living organisms. Currently, the scientific community is concentrating on the pharmacological potential, bioactive combinations, and chemical makeup of different plant species to deliver medications with little adverse effects [2]. Accordingly, metal-based NPs with special biological, electrical, optical, thermal, and characteristics are thought to be crucial for a variety of applications, including those in the fields of health, textile, electronics, energy, space, and the environment [3]. NPs have cytotoxic effects, and their antimicrobial effects have been investigated and proven to be effective [4]. The decrease in the particle size increases the surface area. It possesses antioxidant properties. Antioxidants reduce and eliminate the damage caused due to free radicals [5]. Through the transformation of metallic zinc (Zn) into ZnNPs for improved uses, the developing field of nanotechnology has a significant impact. The use of biological approaches rather than physical and chemical ones for the creation of NPs has increased. ZnNPs, made of metal, have attracted attention in the medical field due to their antibacterial, anticancer, immunomodulatory, sunscreen, and antioxidant properties. As a result, there is a lot of interest in controlling the size of the particle to produce various-sized NPs that are ultrafine and of uniform distribution.

ZnNPs can be created by a variety of procedures either physically or chemically. Wet chemical methods frequently employ harmful chemicals during the synthesis of NPs. Moreover, chemical stabilizers are added, endangering both human health and the environment [6]. However, physical procedures are expensive, and an intense amount of energy is utilized [7]. For the synthesis of ZnNPs, sustainable and environmentally friendly approaches are suggested using biological resources or their extracts as reducing and stabilizing agents. According to reports, NPs created using natural techniques are more biocompatible and bioactive [8]. Attempts to create silver NPs from marine bacteria either by intracellular or extracellular microbial sources are successful [9]. Small creatures living in the aquatic system are known as marine microbes. Extracellular synthesis is preferred over intracellular synthesis because elaborate purifying processes are not involved [10]. From an industrial perspective, marine actinobacteria are one of the most productive families producing pharmaceutically effective secondary metabolites. The unique chemical structures of the bioactive chemicals derived from marine actinobacteria could serve as a foundation for the development of novel pharmaceutical drugs targeted at infections with resistance [11]. Marine microbes are likely the future prospective and considered factories for the production of nanoscale particles by green synthesis [12]. Actinobacteria of the Streptomyces family appear to have considerable antioxidant qualities [13]. Plant species and their extracts are a major component of the green synthesis of ZnNPs. The pharmaceutical sector may find use for the possible antioxidant capabilities of actinobacterium of *Streptomyces *species acquired from seawater or salt water. Researchers have focused their attention on ZnNPs, in the pharmaceutical industry. The biosynthesized ZnNPs were considered to be effective against gram-positive as well as gram-negative bacteria [11-13].

## Materials and methods

Zn sulfate, Yeast Malt Agar, Yeast Malt Broth, and Nutrient agar were purchased from HiMedia and SRL Chemicals Private Ltd. The marine sediment samples were obtained from the Thoothukudi coastal area of Tamil Nadu by Van Veen grab. The sample was well air-dried, and later, 0.1 mg of the sample was dispersed in 10 ml of Tryptic Soy Broth. A 75% concentration of filtered sterilized seawater was employed. The aim was to simulate the ocean’s conditions. The incubation period for the shaker was 24 to 48 hours at 150 RPM and 28 0C. A 1% inoculum was transferred to promote the growth of actual marine bacteria and facilitate gradual enrichment. A fresh medium of liquid culture was developed over 24 hours. The incubation period for the culture was over 48 hours. Serial dilutions were made from the culture grown in Tryptic Soy Broth for 48 hours. To isolate the colonies, 50 L of Tryptic Soy Agar (TSA) was plated. Cyclohexadiene (0.01%) was added as an antifungal agent to 25% of marine water (sterile). Incubation was done at 28°C. ISP-2-International Streptomyces project media or TSA were used to collect and keep the colonies. The plates were then maintained for 14 days at 37°C in an incubator. A thorough method of morphological and biochemical analysis of the properties of the cell wall was required for the polytaxonomical identification of isolated actinobacterial strains. Culture morphology, reverse side, substrate and aerial mycelium color, and melanoid pigment production were among the morphological characteristics used for identification. Biochemical analysis concentrated on sugar patterns and amino acid composition of the cell wall was performed.

Two actinobacteria that produced bioactive secondary metabolites, *Streptomyces *species (MOSEL-ME28) and *Rhodococcus rhodochrous* (MOSEL-ME29), were chosen among the marine sediment-derived isolates for extracellular synthesis of ZnNPs. *Streptomyces *sp. bacteria were grown in a nutrient solution for three days, creating a cell-free broth and harvested cells. Zn sulfate solution was added to the broth, and a control broth without Zn was also prepared. After 24 hours, the broth with Zn was centrifuged to collect the synthesized NPs. The collected NPs were washed, dried, and weighed [14]. 

A loopful of fresh culture was inoculated individually in 100 ml of Tryptic Soy Broth in a titration flask (250 ml) for each bacterial strain. The incubation period was seven days at 30°C and 150 RPM. Centrifugation of the nutrient broth culture was done at 12000 RPM [9]. The supernatant was kept for future research, once the cells had been removed. Before the reaction, the liquid supernatant was filtered via an aseptic 0.22 m filter paper. The NPs obtained from the precursor salt Zn nitrate were examined at 0.5, 1, and 2 micrometers.

A UV-Vis spectrometer measured the light absorption properties of the NPs between 200 and 600 nm. A blank solution without NPs was used for comparison. The NPs were tested against three different bacteria (*Staphylococcus *MRSA,* Klebsiella pneumoniae*, and *Streptococcus mutans*) at two different concentrations (75 μg/mL or 100 μg/mL) . The size of the clear zones around the NPs on agar plates indicated their effectiveness against each bacteria. All results were statistically analyzed using IBM SPSS Statistics for Windows, Version 21.0 (Released 2012; IBM Corp., Armonk, New York, United States), and the graphs were created with the mean ± standard deviation of four replicates.

## Results

**Figure 1:**
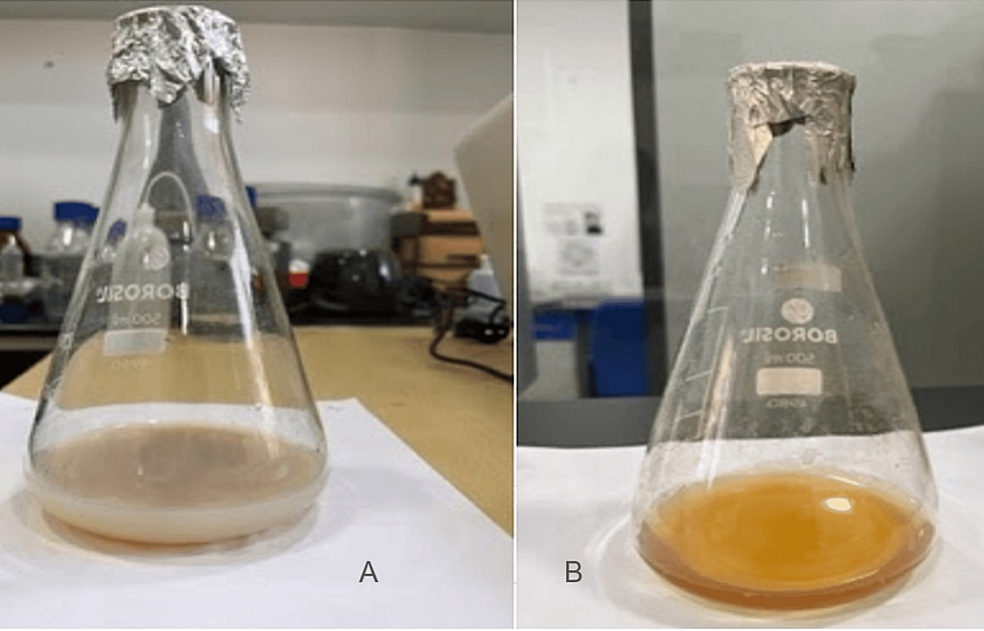
Synthesis of zinc oxide nanoparticle extracts from Actinobacterium Streptomyces species (A) shows a freshly prepared extract of a lighter color, while (B) shows the green synthesis of nanoparticles by the change into a darker color

 Figure 1 shows the change to a darker color indicating the synthesis of Zn oxide (ZnO) NPs after 24 hours.

**Figure 2:**
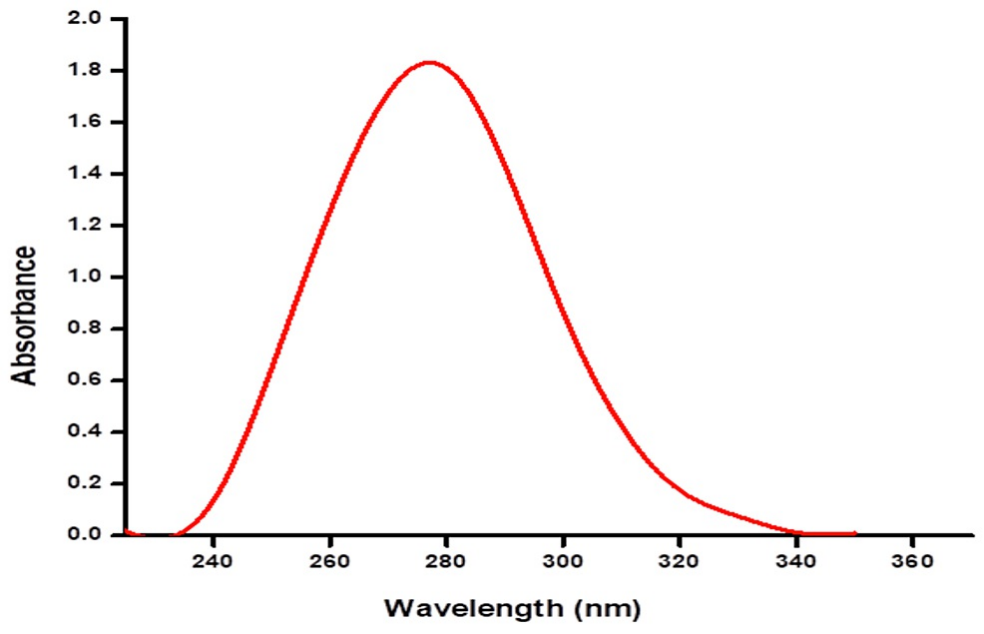
UV spectrum graph indicating the synthesis of zinc oxide nanoparticles with peak absorbance at 280 nm

The UV spectrum graph shows that the ZnONPs are synthesized from Actinobacterium *Streptomyces *species. From the graph, the peak had appeared from 250 to 350 nm. A maximum absorbance of 1.8 was exhibited at a wavelength of 280 nm (Figure 2).

Antibacterial activity 

**Figure 3:**
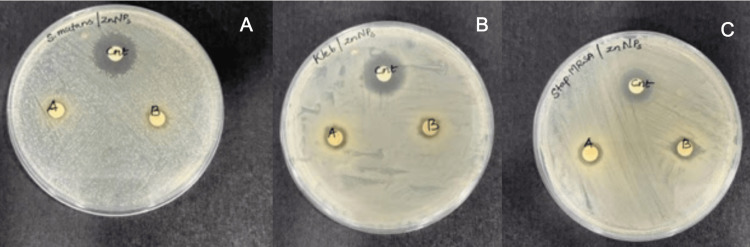
Antibacterial activity In vitro pictures showing antibacterial activity against tested microbial species: A (*Streptococcus mutans*), B (*Klebsiella pneumoniae*), and C (*Staphylococcus *MRSA)

From the experimentation and observation of the sample with bacteria, we found out that with an increase in concentration, there was an increase in the zone of inhibition, seen at the back of the plate. The zone of inhibition of the extract was compared to a standard. At the concentration of 75 μg/mL, the inhibition zones were 8, 8, and 7 mm for *Staphylococcus *MRSA, *Klebsiella pneumoniae*, and *Streptococcus mutans,* respectively. By increasing the concentration by 100 μg/ml, the zone of inhibition was improved to 9.5, 9, and 7.5 mm (Figure 3).

With the increase in concentration, the zone of inhibition was more evidently increased (Table 1).

**Table 1:** Antibacterial activity The mean value of the triplicate experiments done are mentioned

Nanoparticle concentration (µg/ml)	*Staphylococcus aureus* (MRSA) mm	*Klebsiella pneumoniae* (mm)	*Streptococcus mutans* (mm)
75	8	8	7
100	9.5	9	7.5

## Discussion

The synthesis of nanoscale particles in the medical field has a huge global demand. The microorganisms obtained from marine for synthesizing the metal NPs have various positive attributes. A lot of time is saved; it is cost-effective and safe for the environment and a potent antimicrobial agent too [14]. Marine Actinobacteria have been widely researched and proclaimed to produce the drug to cure drug-resistant bacteria and viral infections. The medicine is obtained by remodeling the molecules [15]. Marine organisms such as fungi, yeast, algae, and bacteria can be utilized to produce inorganic NPs both outside or inside the cell [16].

Due to the immense potential of their secondary metabolic products, marine Actinobacteria have been regarded as an untapped treasure trove. Actinobacteria may be used to obtain medications in the future to treat important ailments such as antibiotic-resistant bacteria, cancer, a variety of viral diseases, malaria, and several infestations [17, 18]. Actinobacteria generate a distinct class of biochemical compounds with several carbon skeletons. A fresh wave of increased opportunities is available for the production of medications to act at specific target sites minimizing the side effects and maximizing the efficiency [19]. Antibiotic-resistant microorganisms are a huge concern. Evidence of the potential antimicrobial effects of metal NPs has been found to combat antibiotic resistance. The size of the particle is an important criterion. The medicinal application of NPs obtained from natural resources has minimal adverse effects and is safer [20]. Future research and development will likely increase the demand for novel bioactive chemicals produced by Actinobacteria from a variety of marine sources.

NPs have a greater surface area that enhances the interaction with the pathogenic cells [21]. The selenium NPs obtained from plant resource *Capparis decidua* have proven to have antibacterial activity against* E. coli *and *Lactobacillus *species. While extraction of NPs by conventional chemical methods seems to be hazardous to the environment [22], ecosystem-friendly safe biosynthesis is the best way forward [23]. Silver NPs synthesized from Actinobacteria obtained from the marine ecosystem were proven to be effective in periodontitis. On testing, it proved to suppress infection, reduce inflammation, anti-denaturation effect, and high cytotoxicity [24]. A microtiter-dilution resazurin test was used to assess the antibacterial efficacy of NP alone and in conjunction with antibiotics against multidrug-resistant (MDR) bacteria. It was found to have a synergistic effect [24, 25]. ZnONPs have remarkable and incredible properties. Its dimensions are smaller, have a larger surface area, and are more reactive in nature [26]. There is an alteration in the way they interact with pathogens improving their antimicrobial action. Efforts to understand the mechanics of how they act are an important determinant of their efficacy. NPs are easy to synthesize. The different parameters like size, shape, and ratio can be altered as per application. Silver NPs were effective against *Candida albicans*, *Streptococcus mutans*, *Enterococcus faecalis*, and *Staphylococcus aureus*. There was a significant rise in the zone of inhibition with the increase in the concentration of NPs. As the concentration of the NPs was increased, it caused an evident rise in the inhibition zone [26, 27].

To check the antimicrobial activity of biosynthesized ZnONPs from the marine Actinobacterium, we experimented with three bacteria, namely, *Staphylococcus *MRSA, *Klebsiella pneumoniae, *and *Streptococcus mutans*. The biosynthesized ZnONPs were effective against all of the investigated pathogens. The cytotoxic effects were observed as the concentration was raised. At all concentrations, ZnNPs had significant antibacterial activity [28]. Compared to the standard, the zone of inhibition is good in the case of *Staphylococcus *MRSA(75 μg/ml-8; 100μg/ml-9.5). With *Klebsiella pneumoniae*, the zone of inhibition at 75 μg/ml was 8, and at 100 μg/ml, was 9. In the case of *Streptococcus mutans*, the observed zone of inhibition was 7 at the concentration of 75 μg/ml. By increasing the concentration by 100 μg/ml, the zone of inhibition improved to 7.5. With the increase in concentration, the zone of inhibition was more evidently increased.

The zone of inhibition of the utilized extract could be compared to a standard antimicrobial drug. The widespread use of antibiotics has led to the emergence of microorganisms that are resistant to antibiotics. Hence, the development of novel antibacterial medicines and treatment options to address bacterial infections is imperative [29]. To overcome resistance, larger drug doses of ZnNPs could be administered at the site of infection [30]. Marine-derived ZnNPs could play a pivotal role with their antimicrobial, antioxidant, and anti-cancer potential [31, 32].

The limitation is that the efficiency of NPs differs from species to species, being more efficient on certain strains of the same species. The ZnONPs must be of uniform size, to ensure the stability of the particles and to be universally applicable. Animal studies are needed to confirm the in vivo findings. Our limitations include further analysis of biosynthesized NPs with advanced techniques like diffraction imaging, scanning electron microscopy, and atomic force microscopy. Further in vivo testing will share valuable information about its potential role in the medical field.

## Conclusions

Biosynthesized ZnONPs from the marine Actinobacterium *Streptomyces* species have significant antimicrobial activity against *Staphylococcus *MRSA, *Klebsiella penumoniae*, and *Streptococcus mutans*. The antimicrobial effect increased with the increase in concentrations of extract, and the zone of inhibition was increased. Further in vitro characterization studies and in vivo evaluation will be needed to ascertain the true potential of this novel compound.
